# Polymicrogyria in infants with symptomatic congenital cytomegalovirus at birth is associated with epilepsy: A retrospective, descriptive cohort study

**DOI:** 10.1111/dmcn.16250

**Published:** 2025-01-27

**Authors:** George Lawson, Alexander Sheeka, Pritika Gaur, Styliani Alifieraki, Nigel Basheer, Wajanat Jan, Carolina Kachramanoglou, Hermione Lyall

**Affiliations:** ^1^ Department of Paediatric Infectious Diseases Imperial College Healthcare NHS Trust London UK; ^2^ Department of Infection UCL Great Ormond Street Institute of Child Health London UK; ^3^ Department of Imaging Imperial College Healthcare NHS Trust London UK; ^4^ Department of Surgery and Cancer Imperial College London London UK; ^5^ Department of Paediatrics Imperial College Healthcare NHS Trust London UK; ^6^ Department of Neonatal Medicine Hammersmith Hospital, Imperial College Healthcare NHS Trust London UK

## Abstract

**Aim:**

To identify neonatal magnetic resonance imaging (MRI) features that predict the likelihood of children with congenital cytomegalovirus (cCMV) developing epilepsy, together with clinical features and a validated MRI scoring system.

**Method:**

This was a retrospective descriptive cohort study of infants with cCMV referred to a paediatric infectious disease centre between April 2012 and March 2022, and followed up for at least 2 years. MRI was performed before 4 months of age and assessed by two paediatric neuroradiologists.

**Results:**

Ninety children with cCMV were included, 46 were female and 44 were male. The median age at MRI was 20 days, (standard deviation = 34, range = 1–200). Seventy‐two of 90 children were symptomatic at birth and 7 of 72 developed epilepsy (9.7% of symptomatic infants, 7.8% of total). None of 18 asymptomatic children developed epilepsy. Those with epilepsy were more likely to be symptomatic at birth (100% vs. 76%, *p* = 0.14) and to have cortical malformations (86% vs. 15%, *p* < 0.001). Infants with polymicrogyria (PMG) were more likely to develop epilepsy (odds ratio = 35 [3.9–317.1], *p* < 0.001). A 1‐year remission was achieved in three of seven children; four required multiple antiseizure medications without remission.

**Interpretation:**

The strongest correlate of epilepsy development was PMG. Infants with symptomatic cCMV at birth and PMG were more likely to develop epilepsy, and were likely to require one or more antiseizure medications. Parents of infants with cCMV and cortical malformations should be counselled regarding this risk. Including PMG severity in cCMV MRI scoring could improve epilepsy risk prediction.

## Graphical abstract

In children with symptomatic congenital CMV, those with polymicrogyria are at an increased risk of developing epilepsy.

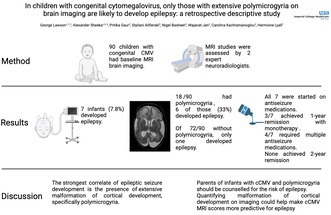

Plain language summary: https://onlinelibrary.wiley.com/doi/10.1111/dmcn.16263

**Abbreviations**
CMV: cytomegalovirus
cCMV: congenital cytomegalovirus
PMG: polymicrogyria

**What this paper adds**

- Epilepsy was observed in 7.8% of children with congenital cytomegalovirus.
- Head circumference was significantly lower in infants with epilepsy.
- Cortical malformation with white matter abnormality was found in all infants with epilepsy.
- Cortical malformation is the sole predictor of epilepsy.

Congenital cytomegalovirus (cCMV) is a leading cause for neurodisability and deafness worldwide. It is the most commonly transmitted virus in utero, with an overall neonatal prevalence of 0.67% (95% confidence interval [CI] = 0.54%–0.83%);^1^ 10% to 15% of infants are characterized as symptomatic at birth, and symptomatic children have a higher risk of sequelae.^2^, ^3^ The recently updated definition for cCMV includes sensorineural hearing loss, meaning that a higher proportion of infants with cCMV will be defined as symptomatic at birth;^4^ 17% to 20% of affected children will develop long‐term sequelae.^5^ Intellectual disability, epilepsy, sensorineural hearing loss, and cerebral palsy are potential neurological sequalae in early life. Few studies have assessed the association between cCMV and epilepsy, and most include low numbers of children. The rate of any seizure is between 7% and 40%;^6^, ^7^, ^8^, ^9^ there is an overall reported rate of 1% to 40% for epilepsy.^10^, ^11^, ^12^

Early transmission of CMV, in the first or second trimester, leads to defective neuronal migration, malformation of cortical development, and neurodevelopmental issues, including epilepsy.^13^, ^14^, ^15^, ^16^

Magnetic resonance imaging (MRI) is the first‐line imaging study for infants with cCMV. Findings reflect the timing of antenatal infection. Earlier infections more often cause polymicrogyria (PMG), cerebellar dysgenesis, and white matter injury with periventricular calcifications and ventriculomegaly. Isolated white matter injury and subependymal cysts are classic of later infection.^17^ PMG, the type of cortical malformation seen in cCMV, is associated with epilepsy, developmental delay, and motor dysfunction.^18^ On MRI, PMG appears as areas of excessive numbers of small, partly fused gyri separated by shallow sulci (Figure 1).^19^

**FIGURE 1:**
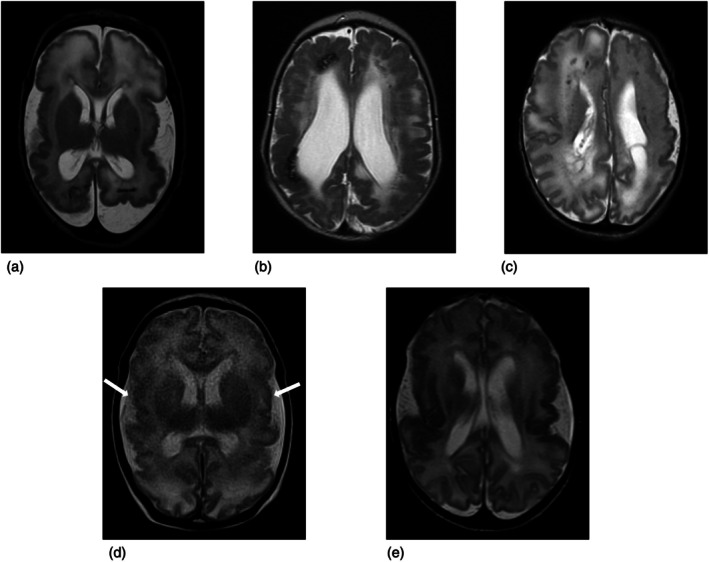
Axial non‐contrast T2‐weighted fast spin echo sequences demonstrating imaging features in a subset of children who developed epilepsy (a,b) and those who did not (c‐e). (a) Two‐month‐old female, born at 30 weeks + 6 days, with diffuse bilateral polymicrogyria (PMG), bilateral caudothalamic groove cysts, bilateral white matter signal abnormality, and parenchymal calcifications in the posterior white matter. She developed epilepsy. (b) One‐day‐old male, born at 38 weeks + 5 days, with diffuse bilateral PMG, bilateral white matter signal abnormality and periventricular parenchymal calcifications in the posterior white matter. He developed epilepsy. (c) Two‐day‐old female, born at 36 weeks + 0 days, with diffuse bilateral PMG, left greater than right, sparing the posterior parietal lobe on the right. She did not develop epilepsy. (d) Twenty‐day‐old male, born at 36 weeks + 5 days, with bilateral perisylvian PMG (white arrows) and patchy white matter signal abnormality. He did not develop epilepsy. (e) Seventeen‐day‐old male, born at 37 weeks + 2 days, with bilateral perisylvian PMG. He did not develop epilepsy.

The incidence of seizures and later development of epilepsy secondary to cCMV infection and related MRI findings, such as structural abnormalities, is not well known. Infants may have MRI changes caused by preterm birth or concurrent pathologies, such as metabolic disturbances, intracranial haemorrhage, and hypoxic‐ischaemic injury. By investigating the development of epilepsy over time and seizure type, and aligning these with baseline neonatal neuroimaging findings, this study aimed to assess which imaging features are associated with epilepsy development in children with cCMV, and to apply the outcome‐validated Alarcon cCMV MRI scoring system (Table 1) as a prognostic indicator.^20^

**TABLE 1:** Postnatal cranial ultrasound and MRI with Alarcon score.^20^

Alarcon score	Findings on postnatal cranial ultrasound and MRI
0	None of the following abnormalities
1	Single punctate periventricular calcification, lenticulostriate vasculopathy, caudothalamic germinolysis, ventriculomegaly (excluding severe), or focal/multifocal white matter signal abnormality on MRI
2	Multiple discrete periventricular calcifications, paraventricular germinolytic cysts, occipital horn septations, severe ventriculomegaly, diffuse white matter signal abnormality, or temporal lobe involvement
3	Extensive calcifications, brain atrophy, abnormal gyration, cortical malformation, dysgenesis of the corpus callosum, or cerebellar hypoplasia

Abbreviation: MRI, magnetic resonance imaging.

## METHOD

We performed a retrospective, descriptive cohort study of infants with cCMV infection referred to the tertiary centre for paediatric infectious diseases at Imperial College Healthcare NHS Trust from April 2012 to March 2022. The centre accepts referrals of children with suspected cCMV from all local hospitals. The study was approved by the ethics review (IRAS project ID: 287554; protocol no.: 20CX6230, Research Ethics Committee reference: 20/HRA/4273). As a retrospective case note review, informed consent was not required for this study.

Children were identified from electronic patient records from the clinic; they were cross‐referenced with a record of patients with suspected CMV on MRI to identify possible missed cases. Infants with confirmed CMV were included, as evidenced by a positive CMV DNA polymerase chain reaction of blood, saliva, or urine in the first 3 weeks of life. They were followed up for at least 2 years in a specialist cCMV clinic at least once per year.

Exclusion criteria included confirmed postnatal CMV infection (often infants born extremely preterm), clinical records unavailable or insufficient, alternative diagnosis, non‐diagnostic or unavailable MRI study, and MRI study performed after 4 months of age.

Clinical data were collected from electronic health records. Routine baseline clinical evaluation included birthweight, birth head circumference, baseline newborn hearing test, detailed clinical examination, ophthalmological examination, blood count, and liver function tests, as well as diagnostic tests for CMV, including infant saliva, urine, or first blood CMV DNA polymerase chain reaction. Infants were retrospectively categorized into those who developed epilepsy and those who did not. All infants were categorized as asymptomatic or symptomatic at birth as per the European consensus criteria (Table 2).^4^ Clinical features included intrauterine growth restriction, microcephaly, petechiae or purpura, neurological signs, and laboratory abnormalities. Infants with any criterion at birth were classified as symptomatic. There were missing data for the assessment of neonatal head circumference, birthweight, and laboratory abnormalities, largely because normal values were often not documented. Those with missing data were excluded for the variable‐specific calculations but remained in the study for the remaining analyses.

**TABLE 2:** Symptoms and signs of infants with cCMV according to the 2024 European consensus criteria.^4^

2024 European consensus criteria
**Clinical symptoms and signs on physical examination**
Intrauterine growth restriction (birthweight ≤2SD for gestational age)
Microcephaly (head circumference ≤2SD for gestational age)
Petechiae or purpura
Blueberry muffin rash (intradermal haematopoiesis)
Jaundice
Hepatomegaly
Splenomegaly
Abnormal neurological examination (lethargy, hypotonia, seizures, poor sucking)
**Abnormal laboratory results**
Anaemia (according to reference haemoglobin and haematocrit values for age and sex)
Thrombocytopenia (<100 000 per μl)
Leukopenia, isolated neutropenia (<1000 per μl)
Elevated liver enzymes (alanine aminotransferase/aspartate aminotransferase at least twice the upper limit of normal)
Conjugated hyperbilirubinaemia (direct bilirubin >2 mg/dL)
**Cerebrospinal fluid**
Abnormal indices, positive CMV DNA
**Neuroimaging**
Abnormalities can be classified into two types: Inflammatory or destructive changes resulting from the direct effect of the virus or the immunoinflammatory response: lenticulostriate vasculopathy, germinolytic pseudocysts (caudothalamic, temporal, frontal), occipital horn septation, ventriculomegaly, periventricular calcifications, white matter abnormalities (i.e. increased signal intensity on T2‐weighted MRI). Brain developmental disruptions: cortical malformations (typically polymicrogyria or poorly developed sulcation), cerebellar hypoplasia.
**Hearing evaluation**
SNHL (hearing threshold > 20 dB, unilateral or bilateral)
**Ophthalmological evaluation**
Chorioretinitis or scarring

Abbreviations: CMV, cytomegalovirus; cCMV, congenital CMV; MRI, magnetic resonance imaging; SNHL, sensorineural hearing loss.

All brain MRI examinations were retrospectively reviewed by two expert consultant neuroradiologists who were aware that infants were investigated for cCMV infection. MRI examinations were performed at several units, using different scanners, field strength, and magnetic resonance protocols. Consequently, acquisition protocols varied. Almost all studies included axial T2‐weighted and axial T1‐weighted diffusion‐weighted imaging and a blood‐sensitive sequence (either susceptibility‐weighted sequence or gradient echo). Where a blood‐sensitive sequence was absent, the B0 map of the diffusion‐weighted imaging sequence was used to assess the presence of calcifications. B0 is the ‘baseline’ image with no diffusion attenuation.

Neuroimaging results were categorized as normal or abnormal based on the presence or absence of magnetic resonance abnormalities known to suggest cCMV infection. Each MRI examination was scored using the validated imaging score for infants with cCMV, devised by Alarcon et al. (Table 1).^20^

### Statistical analysis

A Fisher's exact test was used to determine if a significant association existed between imaging findings (independently and on the Alarcon scale) and the presence of seizures. A Student's *t*‐test was performed to assess the variation in the mean birthweight and head circumference of both groups (with and without epilepsy) based on standard z‐scores. A *χ* ^2^ test was performed to assess the relationship between the specific pattern of PMG and seizure likelihood. Statistical significance was determined as *p* = 0.05. Missing values were managed by omission of the participant for analysis of that variable. Missing MRI data were one of the exclusion criteria. All statistics were performed using MATLAB (MathWorks, Natick, MA, USA).

## RESULTS

Ninety‐eight infants were identified for the study. Eight were excluded, leaving 90 for the final analysis. Exclusions were as follows: four lacked adequate clinical data; three lacked adequate MRI studies; and one was diagnosed with *CNPY3* epileptic encephalopathy. Figure S1 includes a cohort diagram.

Of the infants included in the final analysis, 46 were female (51.1%), 69 (76.7%) were born at term, 21 (23.3%) were born preterm (<37 weeks gestation), and 72 (80%) were classified as symptomatic at birth. The most frequent symptoms were sensorineural hearing loss (38 of 90, 42.2%) and intrauterine growth restriction (36 of 90, 40%). Neurological features were present in 12 infants (13.3%), usually as a combination of features. One infant had neonatal seizures secondary to hypoxic‐ischaemic encephalopathy based on clinical and radiological examination, and required no further treatment. The most frequent laboratory abnormality was thrombocytopenia, reported in 11 infants (12.2%). However, there were only 16 laboratory abnormalities, with 78 children having no values documented. The median duration of the follow‐up was 8 years for those who developed epilepsy (range = 6–12 years) and 6 years (range = 2–16 years) for those who did not. Seizures were reported in eight children (8.9%). As mentioned earlier, one infant had isolated seizures at birth symptomatic of hypoxia‐ischaemia and was therefore analysed with the group without epilepsy. Seven children (7.8%) developed epilepsy and their seizure type, treatment, and outcomes correlating with the MRI findings are shown in Table 3.

**TABLE 3:** Seizure type, epilepsy syndrome, and outcomes in children with congenital CMV who developed epilepsy.

Age at first seizure, months	Seizure type	Antiseizure medication(s)	Treatment response		Mobility
			1‐year remission	2‐year remission	
20	Focal, subclinical, nocturnal seizures, electrographic only status of sleep	Levetiracetam	Yes	No	GMFCS level V
11	Focal tonic, autonomic, awareness not always lost	Levetiracetam + valproate	No	No	Walks with limitations
15	Non‐convulsive, myoclonic, bilateral tonic	Levetiracetam + lamotrigine	No	No	GMFCS level V
19	Non‐convulsive, bilateral tonic (daily)	Carbamazepine + levetiracetam + valproate	No	No	Not walking, limited other mobility
48	Non‐convulsive, bilateral, tonic–clonic, nocturnal	Oxcarbazepine (switched from carbamazepine), previously levetiracetam	Yes	No	Walks with limitations
18	Bilateral tonic–clonic, nocturnal	Carbamazepine	Yes	No	Walks with limitations
6	Bilateral tonic, nocturnal	Levetiracetam + valproate	No	No	Not walking, limited other mobility

Abbreviations: CMV, cytomegalovirus; GMFCS, Gross Motor Function Classification System.

### Seizure type and management

The median age at first epileptic seizure was 18 months (range = 6–48 months). Of the seven children with epilepsy, two had their first seizure before 12 months of age; none had seizures during the neonatal period. Multiple seizure types were observed, including focal tonic, bilateral tonic–clonic, myoclonic, and non‐convulsive seizures (atypical absences and subclinical seizures and electrical status epilepticus of sleep). All children had focal seizures; six suffered focal impaired awareness seizures, and bilateral tonic–clonic/tonic seizures were observed in five. Non‐convulsive seizures were seen in six (two with atypical absences, one with myoclonic seizures, two with autonomic features, and one subclinical) and four had nocturnal seizures (one was subclinical and one had electrographic only status epilepticus in sleep). None had infantile spasms. Electroencephalogram patterns varied from focal to bilateral multifocal epileptiform discharges that were accentuated in sleep, and backgrounds that included normal sleep architecture to poorly defined posterior rhythms and asymmetries. All seven infants initiated antiseizure medications. Three children achieved 1‐year remission with monotherapy. The other four required two or more antiseizure medications and none achieved seizure remission, with one suffering daily seizures.

### Clinical findings at birth

Of the seven children with epilepsy, three (42.9%) were born preterm compared to 18 of 83 (21.7%) children in the group without epilepsy. All of the children who developed epilepsy were classified as symptomatic at birth. Most in the group without epilepsy were also symptomatic (65 of 83, 78.3%).

Microcephaly was documented in five of the seven children who developed epilepsy (71.4%), compared to 12 of 83 (14.5%) who did not. A specific value for head circumference at birth was only recorded in three children from the group with epilepsy and 48 from the group without, with 38 children excluded for this specific assessment. The mean head circumference of the epilepsy group was significantly lower (z‐score − 3.07 vs. −1.54, *p* = 0.04). Microcephaly is defined as a z‐score less than −2. A smaller head circumference was significantly associated with developing epilepsy (*p* = 0.012).

In the group with epilepsy, three had a recorded birthweight; in the cohort without epilepsy, 72 of 83 had a recorded value. There was no significant difference in mean birthweight (z‐score − 1.45 vs. −1.37, *p* = 0.91).

### Imaging findings

The median age at MRI was 20 days of life (standard deviation = 34, range = 1–200); the median gestational age was 41 weeks plus 4 days. In the seven infants with epilepsy, imaging demonstrated PMG in six (85.7%), cyst formation in six (85.7%), and white matter calcifications in four (57.1%) infants. These were more prevalent than in the group without epilepsy, with PMG in 12 of 83 (14.5%), cyst formation in 37 of 83 (44.6%), and calcifications in 10 of 83 (12%). PMG was the only type of malformation of cortical development identified.

Of all 18 infants with PMG, six (33.3%) developed epilepsy. Of 72 infants without PMG, only one developed epilepsy (1.4%). There was a statistically significant association between epilepsy and the presence of PMG, with an odds ratio of 35.5 (95% CI = 3.9–317.1, *p* < 0.001). In most infants (4 of 6) with PMG and epilepsy, the distribution of PMG was diffuse (involving the entire hemisphere or all lobes with spared areas) and bilateral. Of the remaining two children, one had unilateral right hemispheric PMG and the other had asymmetric (right more than left), predominately perisylvian PMG. Of the infants who did not develop epilepsy, diffuse bilateral PMG was seen in 6 of 82 (7.3%); one demonstrated unilateral right hemispheric PMG, three bilateral temporal PMG, one bilateral perisylvian PMG, and one bilateral frontotemporal PMG. On review of imaging, there were no apparent discriminating radiological factors that appeared to predict epilepsy risk in infants with extensive PMG. There was no statistically significant link between the distribution of PMG and the development of epilepsy (*p* = 0.42).

Of the seven infants who developed epilepsy, six (85.7%) had an Alarcon brain imaging score of 3, compared to only 12 of 83 (14.5%) of those who did not develop epilepsy. The one infant with epilepsy without a cortical malformation and an Alarcon score of 2 was born preterm, had other congenital anomalies (eye coloboma), as well as microcephaly and intrauterine growth restriction, and was suspected of having a genetic epileptic syndrome, although none has been found so far. MRI demonstrated extensive white matter changes only.

There was an association with calcifications and epilepsy (*p* < 0.01). An association was seen with white matter abnormality and epilepsy (*p* = 0.04). However, when asymptomatic patients were excluded from the analysis, a statistical relationship only remained with PMG (*p* < 0.001) and calcifications (*p* = 0.02).

## DISCUSSION

This is the largest study looking specifically at the association between baseline neuroradiological findings and epilepsy in children with cCMV. In our cohort of 90 children with cCMV, where 72 (80%) were symptomatic at birth, only seven (7.8%) developed epilepsy. All of the children who developed epilepsy were symptomatic at birth.

In the group of seven with epilepsy, the median age at first seizure was 18 months; two children had a seizure before they were 1 year old. Focal tonic or bilateral tonic–clonic seizures were seen in all children; non‐convulsive nocturnal electrographic only seizures were observed in two children, none had infantile spasms. There was a high incidence of non‐responsiveness to antiseizure medication: three children achieved 1‐year remission with monotherapy; four children required two or more antiseizure medications with none achieving 1‐year remission; no child achieved a 2‐year remission.

Traditionally, a strong link between the presence of intracranial calcifications and seizure development has been observed.^21^ However, new insights have suggested that intracranial calcifications are typically associated with injury to the white matter. Therefore, they are not expected to be present in the absence of white matter signal abnormality; other features, including migration disorders, are more predictive of epilepsy.^10^, ^20^, ^22^, ^23^, ^24^

In this large cohort with full MRI, the strongest correlate of epileptic seizure development was the presence of extensive PMG (*p* < 0.001); PMG was the only cortical malformation abnormality. Cortical malformation abnormalities were more common in the group with seizures (85.7%) compared to the group without (14.5%). Additionally, the distribution of PMG was more often diffuse or global in the group with seizures. In the literature, malformations of cortical development are a common cause of medically non‐responsive epilepsy, particularly in children.^25^ On review of imaging, there were no apparent discriminating radiological factors to predict epilepsy risk in the small number of infants with extensive PMG; it cannot be easily explained why some infants with widespread PMG did not develop epilepsy. On the other hand, only one infant without extensive PMG developed epilepsy and they had other congenital abnormalities suggestive of an alternative systemic cause. Other studies specifically looking at PMG have also shown a high rate of epilepsy (78%).^26^ Like our study, there was no observed correlation between the specific location of PMG and epilepsy subtypes.

In our study, no child achieved a 2‐year seizure remission. When assessing childhood epilepsies in general, only 15% of children are non‐responsive to pharmacological treatment and around 40% of non‐responsive cohorts demonstrate PMG.^27^ Follow‐up of children in our centre suggests that seizures associated with cCMV and PMG may be difficult to manage on monotherapy. Although 1‐year remissions were achieved in some children (3 of 7), those on multiple medications did not achieve meaningful remission. There was also a substantial risk (4 of 7 children) of significant nocturnal features. This should be considered when counselling parents and directing investigations and treatment.

Second‐trimester infection with CMV is linked with white matter disease.^17^ There are studies confirming the high positive predictive value of the presence of abnormal white matter on MRI in cCMV and adverse neurological outcomes, such as developmental delay.^28^ White matter changes are thought to reflect a cytokine‐mediated fetal immune reaction to a relatively late infection. In our study, there was a correlation between white matter signal abnormalities and seizures. However, this was not significant when asymptomatic children were removed from the analysis. Although white matter signal abnormality was present in all of the children who developed epilepsy, it was always present with malformations of cortical development.

In terms of neonatal clinical predictors in children with cCMV infection, unsurprisingly our study showed that a smaller head circumference at birth was associated with subsequent development of epilepsy. Other baseline clinical features, including gestational age, intrauterine growth restriction, hepatomegaly, splenomegaly, petechiae, and chorioretinitis were not associated with the development of epilepsy.

The presence of global PMG and a high MRI score correlated with the likelihood of developing epilepsy. However, this study did not demonstrate that the MRI scoring system could independently predict epilepsy risk. Review of the scoring system to include a quantification of the extent and severity of PMG could make this more predictive. Beyond classification at birth (symptomatic or asymptomatic), a future baseline neonatal scoring system for cCMV that can prognosticate for children more effectively should include MRI, and we specifically highlight the significance of PMG. On the other hand, based on the findings of this study, we suggest that neonatal seizures should not be included. The children who developed epilepsy in our cohort did not present with neonatal seizures; indeed, the child with neonatal seizures had an insult not related to cCMV. This should be considered by CCMVnet, which is developing a more predictive neonatal scoring system from a data set of more than 1500 children with cCMV enrolled in the European Registry (https://ccmvnet.org/).

### Limitations

This is a single‐centre, clinical, retrospective, descriptive study. Despite including a single centre, it is the largest study looking specifically at the association between cCMV and epilepsy and receives referrals from the region; it is likely to be representative of other European urban populations. Second, missing data were an issue, including baseline birth data such as head circumference, and laboratory abnormalities because normal values were often not documented and tests performed at the referring centres were unavailable. However, these missing data would not be likely to affect the conclusions based on the radiographic findings. Third, children may develop seizures after the follow‐up. However, this has not been commonly observed; in our study, where the median follow‐up was 6 years, no child developed seizures after 48 months and most by 24 months. Fourth, the findings on MRI may be affected by image quality, for example, small areas of PMG may be difficult to appreciate. Finally, the inferences made are based on small numbers and this highlights the need for larger prospective cohorts, like those being collected via the European Registry.

### Conclusions

A combination of extensive cortical malformation with white matter abnormality was found in all but one of the infants with cCMV who developed epilepsy. The presence of PMG conferred 35.5 times higher odds of developing epilepsy. Clinicians and parents caring for infants with symptomatic cCMV with these features should be made aware of the significant risk of their children developing epilepsy; parents should also receive prospective training in seizure management.

## FUNDING INFORMATION

There was no specific funding for this study.

## CONFLICT OF INTEREST STATEMENT

The authors of this manuscript have no conflicts of interest to declare.

## Supporting information


**Figure S1:** Cohort diagram describing exclusions and the final breakdown of included children based on symptom and epilepsy status.

## Data Availability

The data that support the findings of this study are available on request from the corresponding author. The data are not publicly available due to privacy or ethical restrictions.
