# Supplementary material for: Polymicrogyria in infants with symptomatic congenital cytomegalovirus at birth is associated with epilepsy: A retrospective, descriptive cohort study
Source: Dev Med Child Neurol. 2025 Jan 27;67(8):1026–33. doi: 10.1111/dmcn.16250 (PMC12237226; doi:10.1111/dmcn.16250)
Supplement: Supplementary file 1 — Figure S1: Cohort diagram describing exclusions and the final breakdown of included children based on symptom and epilepsy status. [file DMCN-67-1026-s001.docx]

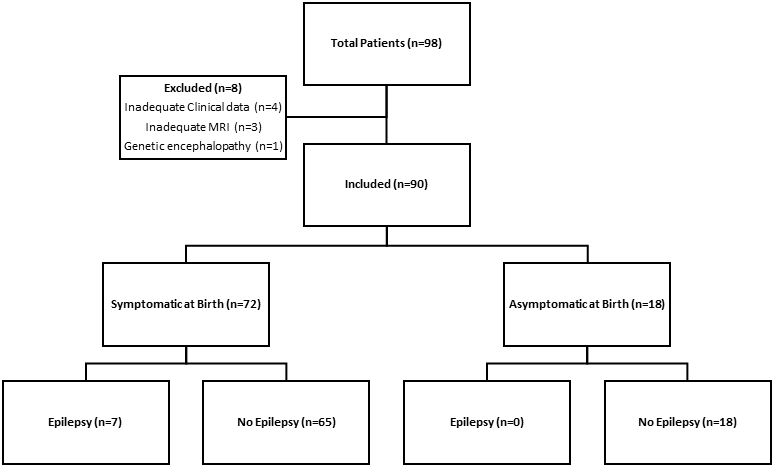


**Figure 2:** Cohort diagram describing exclusions and the final breakdown of included patients based on symptom and epilepsy status. Table describing symptoms, Alarcon score and MRI findings in each patient. Denominator is number of children in each group, except for Number where the denominator is all children.

* 1 patient developed neonatal seizures which did not recur. This patient was included in the no epilepsy cohort.

|  | **All Children** | | **Symptomatic Epilepsy** | | **Symptomatic No Epilepsy** | | **Asymptomatic No Epilepsy** | |
| --- | --- | --- | --- | --- | --- | --- | --- | --- |
| **Number** | 90 | *100%* | 7/90 | *7.8%* | 65/90 | *62.2%* | 18/90 | *20%* |
| **Female** | 46 | *51.1%* | 5 | *71.4%* | 33 | *50.8%* | 8 | *44.4%* |
| **Median gestational age (weeks)** | 38+2 |  | 37+5 |  | 38 |  | 39 |  |
| **Premature (<37 weeks)** | 21 | *23.3%* | 3 | *42.9%* | 17 | *26.2%* | 1 | *5.6%* |
| **Median Head circumference (z-score)** | -1.64 | | -3.07 | | -1.81 | | -0.55 | |
| **Median Birth Weight (z-score)** | -1.39 | | -1.45 | | -1.74 | | -0.33 | |
| **Any symptom or sign** | 72 | *80%* | 7 | *100%* | 65 | *100%* | 0 |  |
| **SNHL** | 38 | *42.2%* | 4 | *57.1%* | 34 | *52.3%* | 0 |  |
| **IUGR** | 36 | *40%* | 4 | *57.1%* | 32 | *49.2%* | 0 |  |
| **Microcephaly** | 17 | *18.9%* | 5 | *71.4%* | 12 | *18.5%* | 0 |  |
| **Petechiae/purpura** | 14 | *15.6%* | 3 | *42.9%* | 11 | *16.9%* | 0 |  |
| **Blueberry muffin rash** | 3 | *3.3%* | 1 | *14.3%* | 2 | *3.1%* | 0 |  |
| **Jaundice** | 8 | *8.9%* | 2 | *28.6%* | 6 | *9.2%* | 0 |  |
| **Hepatomegaly or splenomegaly** | 5 | *5.6%* | 1 | *14.3%* | 4 | *6.2%* | 0 |  |
| **Lethargy** | 11 | *12.2%* | 4 | *57.1%* | 7 | *10.8%* | 0 |  |
| **Hypotonia** | 14 | *15.6%* | 3 | *42.9%* | 11 | *16.9%* | 0 |  |
| **Poor suck** | 12 | *13.3%* | 3 | *42.9%* | 9 | *13.8%* | 0 |  |
| **Seizure** | 1 | *1.1%* | 0 | *0%* | 1 | *1.5%* | 0 |  |
| **Anaemia** | 1 | *1.1%* | 0 | *0%* | 1 | *1.5%* | 0 |  |
| **Neutropaenia** | 3 | *3.3%* | 1 | *14.3%* | 2 | *3.1%* | 0 |  |
| **Thrombocytopaenia** | 11 | *12.2%* | 2 | *28.6%* | 9 | *13.8%* | 0 |  |
| **Hyperbilirubinaemia** | 1 | *1.1%* | 0 | *0%* | 1 | *1.5%* | 0 |  |
| **Elevated AST/ALT** | 0 | *0%* | 0 | *0%* | 0 | *0%* | 0 |  |
| **Retinitis** | 4 | *4.4%* | 3 | *42.9%* | 1 | *1.5%* | 0 |  |
| **Retinal scarring** | 1 | *1.1%* | 0 | *0%* | 1 | *1.5%* | 0 |  |
| **Alarcon Score** |  |  |  |  |  |  |  |  |
| **0** | 31 | *34.4%* | 0 |  | 20 | *30.8%* | 11 | *61.1%* |
| **1** | 14 | *15.6%* | 0 |  | 12 | *18.5%* | 2 | *11.1%* |
| **2** | 27 | *30%* | 1 | *14.3%* | 22 | *33.8%* | 4 | *22.2%* |
| **3** | 18 | *20%* | 6 | *85.7%* | 11 | *16.9%* | 1 | *5.6%* |
| **MRI findings** |  |  |  |  |  |  |  |  |
| **Calcifications** | 14 | *15.6%* | 4 | *57.1%* | 9 | *13.8%* | 1 | *5.6%* |
| **Supependymal Cysts** | 43 | *47.8%* | 6 | *85.7%* | 33 | *50.8%* | 4 | *22.2%* |
| **White Matter Changes** | 53 | *58.9%* | 7 | *100%* | 40 | *61.5%* | 6 | *33.3%* |
| **Malformation of Cortical**  **Development / Polymicrogyria** | 18 | *20%* | 6 | *85.7%* | 11 | *16.9%* | 1 | *5.6%* |
